# Carbon Quantum Dots Conjugated Rhodium Nanoparticles as Hybrid Multimodal Contrast Agents

**DOI:** 10.3390/nano11092165

**Published:** 2021-08-24

**Authors:** Giovanni M. Saladino, Nuzhet I. Kilic, Bertha Brodin, Bejan Hamawandi, Idris Yazgan, Hans M. Hertz, Muhammet S. Toprak

**Affiliations:** 1 Department of Applied Physics, Biomedical and X-ray Physics, KTH Royal Institute of Technology, SE-10691 Stockholm, Sweden; nikilic@kth.se (N.I.K.); berthab@kth.se (B.B.); bejan@kth.se (B.H.); hans.hertz@biox.kth.se (H.M.H.); 2 Center of Biosensors and Materials, Department of Biology, Faculty of Science and Arts, Kastamonu University, Kastamonu 37150, Turkey; iyazgan@kastamonu.edu.tr

**Keywords:** X-ray fluorescence, carbon quantum dots, contrast agents, dual-mode imaging, nanomedicine, hybrid nanostructure, bio-imaging

## Abstract

Nanoparticle (NP)-based contrast agents enabling different imaging modalities are sought for non-invasive bio-diagnostics. A hybrid material, combining optical and X-ray fluorescence is presented as a bioimaging contrast agent. Core NPs based on metallic rhodium (Rh) have been demonstrated to be potential X-ray Fluorescence Computed Tomography (XFCT) contrast agents. Microwave-assisted hydrothermal method is used for NP synthesis, yielding large-scale NPs within a significantly short reaction time. Rh NP synthesis is performed by using a custom designed sugar ligand (LODAN), constituting a strong reducing agent in aqueous solution, which yields NPs with primary amines as surface functional groups. The amino groups on Rh NPs are used to directly conjugate excitation-independent nitrogen-doped carbon quantum dots (CQDs), which are synthesized through citrate pyrolysis in ammonia solution. CQDs provided the Rh NPs with optical fluorescence properties and improved their biocompatibility, as demonstrated in vitro by Real-Time Cell Analysis (RTCA) on a macrophage cell line (RAW 264.7). The multimodal characteristics of the hybrid NPs are confirmed with confocal microscopy, and X-ray Fluorescence (XRF) phantom experiments.

## 1. Introduction

Bio- and medical imaging have been extensively used due to their visual interface with the goal of enabling early detection and diagnosis of diseases. It is, therefore, essential to develop supplements that can maximize the ability of the current detection schemes, diagnosis, and treatment of variety of diseases, such as cancer [1]. Nanoparticles (NPs) are presently widely investigated as carriers for targeted drug delivery and therapy [2,3]. NP-based contrast agents have been the subject of intensive research. One of the most attractive features of NPs is the possibility of modulating their bio-distribution by surface derivatization, or conjugation with agents for specific targeting. Synthetic control over the size and morphology of NP contrast agents can significantly influence pharmacokinetics and biodistribution, and surface modification enables therapeutic efficacy and multimodal imaging [4]. There are commercial contrast agents, which are viable for preclinical or clinical imaging [5,6]. Different bioimaging techniques require the use of specific contrast agents. Computed tomography (CT), for example, uses iodine-based small molecules and barium suspensions. Magnetic resonance imaging (MRI) utilizes superparamagnetic iron oxide NPs and gadolinium chelates. Quantum dots, gold, and rare earth oxide NPs are used in optical imaging, while gold nanoparticles are also utilized for photoacoustic imaging. Ultrasound imaging makes use of silica (SiO_2_) NPs and gas-filled microbubbles. Lastly, nuclear imaging (PET, SPECT) techniques use radionuclide-labeled compounds [1].

Laboratory NP X-ray fluorescence tomography is an emerging biomedical imaging modality with potential for high-spatial-resolution molecular small-animal imaging [7,8]. X-ray fluorescent NPs have been successfully employed in bioimaging, to reveal biophysical characteristics and features in cellular environments [8,9]. Recently, we have demonstrated the use of NP-based contrast agents for X-ray fluorescence computed tomography (XFCT) in preclinical research and for tumor detection using MoO_2_ NPs, utilizing a liquid-metal-jet microfocus source [8,10]. Furthermore, we also validated the potential use of Rh- and Ru-based NPs as XFCT contrast agents, which have matching X-ray absorption profiles to the pencil beam X-ray source [11,12,13]. In order to improve the functionality and biocompatibility of the XFCT contrast agents, SiO_2_ coating was introduced, where a fluorophore (Cy5.5) has been covalently attached to the SiO_2_ matrix. The synthesized core-shell NPs were then validated for in vitro and in situ bioimaging [14].

For the synthesis of metallic NPs, there are several methods reported in the literature; in particular, wet-chemistry-mediated synthesis of metallic nanostructures are of great interest owing to their flexible design in controlling the size, shape, and surface chemistry [15]. Chemical reduction [16], UV photolysis, thermal decomposition [17], metal vapor deposition [18], electrochemical reduction [19], sonochemical decomposition [20], and microwave irradiation [21] are among the commonly used methods. Among these, the chemical reduction method is a rapid and scalable route to prepare water-dispersible NPs. The process can be done in an aqueous solution using reducing agents like NaBH_4_, or in long-chain alcohol media (also known as polyol synthesis), where the alcohol acts as the reducing agent and solvent at the same time. Rh nanostructures, including nanoshells, nanoframes and porous nanoplates, were recently reported through an inverse-directional galvanic replacement reaction [22]. Rh NPs synthesis was also reported through polyol reduction [11,12]. This approach, and other classical approaches, require multiple steps including synthesis, activation, isolation, and functionalization. These steps cause increased byproduct formation, toxic chemical utilization while limiting large scale, inexpensive, and benign production accompanied by high reproducibility [23]. Therefore, methods free of toxic byproducts with minimal process steps are under intensive investigation. Biomolecules including amino acids, peptides [24,25], sugar ligands, and sugar polymers [26] have been shown to act as reducing, capping, and stabilizing agents in the synthesis of metallic and ceramic nanostructures [27,28,29,30].

In recent years, carbon quantum dots (CQDs) have gained significant attention [31,32,33,34]. Possessing superior properties including inertness, low toxicity, large surface area, high photostability, high resistance to photobleaching, and easy surface modification make them promising materials for biomedical applications [35,36,37,38]. Doped CQDs can be a versatile material for future biomedical and sensing applications due to their tunable fluorescent properties, excellent biocompatibility, and high aqueous stability [39]. There has been intensive research, using several heteroatoms (nitrogen, sulfur, phosphorus, boron, fluorine, etc.) as well as metals (Zn, Mg, Ag, Au, Cu, Ga, etc.) as doping agents to enhance the physicochemical properties of CQDs [40,41,42].

In this work we report the synthesis and functional analysis of Rh-CQD hybrid NPs, where a specially designed sugar ligand is used as a reducing and capping agent during the aqueous synthesis of Rh NPs. CQDs were synthesized through MW-assisted pyrolysis and thereafter have been conjugated to the Rh NPs’ surface. This conjugation conferred Rh NPs with optical fluorescence properties and improved biocompatibility. The dual mode properties of the Rh-CQD hybrid NPs were confirmed via confocal microscopy for in vitro localization, and with XRF phantom experiments, to mimic in vivo imaging.

## 2. Materials and Methods

*Materials*: Rhodium(III) chloride (RhCl_3_, 98%), Citric acid (≥99.5%), N-Hydroxysuccinimide (NHS, ≥97%), N-(3-Dimethylaminopropyl)-N’-ethylcarbodiimide hydrochloride (EDC, BioXtra), Lactose (100%), 4,4′-Oxydianiline (97%), Borane dimethylamine complex (97%), Ammonia solution (25%), Quinine hemisulfate salt monohydrate (≥99%), Sulfuric acid (≥97%), Acetic acid (≥99%), Acetone (≥99.5%), Iscove’s Modified Dulbecco’s Medium (IMDM), Fetal Bovine Serum (FBS), and Murine macrophages (RAW 264.7, 91062702-1VL) were all purchased from Sigma Aldrich (Stockholm, Sweden). Phosphate-Buffered Saline (PBS) and the fluorescent probe, Alexa Fluor 555 Phalloidin, were purchased from Thermo Fisher Scientific (Stockholm, Sweden). Ethanol absolute (≥99.8%) was purchased from VWR International AB (Stockholm, Sweden).

*Sugar Ligand*: Lactose 4,4′-oxydianiline-N (LODAN) sugar ligand was obtained with a two-step reductive amination reaction in 50:50 acetic acid:water solvent system, as described in previous studies [30,43,44]. Shortly, a molar ratio of 1.2 between amino groups in the organic substituents to the sugar moieties was chosen in order to fully deplete the sugar content, allowing the precipitation of the final product, while washing away the residual organic groups with acetone and ethanol rinse. Borane dimethylamine complex was used to convert imine intermediate into amine, in a ratio of 1.2 with the sugar residue. The sugar ligand was then dried at 70 °C in a rotavapor for further use.

*Rhodium Nanoparticles*: Rh NPs were synthesized via a Microwave (MW)-assisted hydrothermal method. Typically, the Rhodium precursor, RhCl_3_ ([Rh^3+^] = 2 mM), was mixed with LODAN (4 mM) in 15 mL water at RT, under magnetic stirring. The dispersion was processed for 5 min at 160 °C with MW irradiation at 2.45 GHz in the Initiator + SP Wave (Biotage^®^, Uppsala, Sweden), turning the dispersion from light yellow to dark black color. After cooling down, the dispersion was transferred into dialysis centrifuge tubes (30 kDa) and centrifuged to remove unreacted precursors. The collected NPs were then re-dispersed in distilled water.

*Carbon Quantum Dots*: For the synthesis of CQDs, a MW-assisted hydrothermal method was pursued. In a typical reaction, citric acid (260 mM) was dissolved in ammonia (30 mL) under continuous stirring. The solution was then transferred to a Teflon for MW treatment in the flexi-WAVE (Milestone SRL, 24010 Sorisole, Bergamo, Italy) at 200 °C for 30 min. The sample was then transferred into a beaker and diluted with water (40 mL). The ammonia solution was evaporated at 60 °C, while magnetically stirring, until the dispersion reached a neutral pH; the solution turned its color from light yellow to dark blue. The synthesized CQDs were stored in the refrigerator, in a dark environment.

*Conjugation Process*: Rh NPs were conjugated with CQDs by cross-linking the amino groups on the Rh NPs’ surface to the carboxyl groups of the CQDs. Stock solutions of NHS and EDS were prepared, with a concentration of 1 mg/mL. Under magnetic stirring, to a dispersion of CQDs (60 μg/mL), NHS and EDC were introduced, with a final concentration of 200 μg/mL. Subsequently, Rh NPs (0.6 mM) were added into the dispersion and reacted for 24 h in a sealed beaker at room temperature. Finally, the dispersion was centrifuged, and the precipitate was re-dispersed in milli-Q water. The Rh-CQDs NPs were stored at 4 °C, in dark, for further use.

*Characterization Techniques*: Ultraviolet−Visible Spectrophotometry (UV−vis, NP80, Implen) was used for the determination of the absorption spectrum. A spectrofluorometer (Jasco FP-8300, Kovalent AB, Västra Frölunda, Sweden) was employed to evaluate the optical fluorescence properties of the synthesized samples, diluted in milli-Q water. For the estimation of the relative fluorescence Quantum Yield (QY), quinine hemisulfate salt monohydrate (10 μg/mL) was dissolved in sulfuric acid (0.5 M) and the fluorescence and absorption spectra recorded. Further details are presented in the Supplementary Materials. The hydrodynamic size and surface charge (zeta potential) of diluted dispersions (pH 6.5) of the synthesized stocks were measured with the Zetasizer Nano ZS90 (Malvern Panalytical Ltd., Malvern, UK), in triplicates. The size and morphology of NPs (in dried state) were estimated via Transmission Electron Microscopy (TEM, JEM-2100F, JEOL Ltd., Tokyo, Japan). Energy Dispersive X-ray Spectrometry (EDS) was used to record the energy spectrum of the prepared TEM samples. Infrared spectra of different samples were recorded with FT-IR Spectroscopy (Thermo Fisher Scientific, Stockholm, Sweden), to identify the vibrational peaks of specific functional groups. Thermogravimetric Analysis (TGA) was utilized for composition analyses, with a TGA550 (TA Instruments Sweden, Solna, Sweden). The concentration of CQDs was estimated through the evaporation of 1 mL from the stock solution (in triplicates). The water content on the dried samples was estimated via TGA, and its percentage was removed from the overall weight. X-ray fluorescence (XRF) measurements on the sample stocks were used to estimate the concentration of Rhodium in Rh NPs and Rh-CQDs NPs.

*Cytotoxicity Assay*: Real-time Cell Analysis (RTCA, xCELLigence, Agilent Technologies Sweden AB, Sundbyberg, Sweden) was used to monitor the behavioral changes of the RAW 264.7 (91062702-1VL, Sigma Aldrich, Stockholm, Sweden) adherent cells in real-time. A total of 7000 murine macrophage cells were added into each well and let to adhere to the bottom of the wells overnight. The medium was subsequently removed and replaced with a dispersion containing medium and the corresponding concentration of NPs, with a total volume of 100 μL. The impedance measurements were made on triplicates, every 4 h, to follow the behavioral changes as a function of time. The arbitrary quantity, Cell Index (CI), was normalized at the time of NPs exposure. Untreated cells were used as control and exposed only to the medium.

*Confocal Optical Imaging*: 20,000 cells were cultured in confocal chamber wells, following the same procedure as in the cytotoxicity assay with the same procedure as RTCA. The medium including the NPs was kept for 3 or 24 h, then removed and replaced by PBS. The cells were then fixed with formaldehyde and dried with methanol. Finally, the cells were stained with Alexa Phalloidin 555, to bind the actin filaments and highlight the cell morphology.

*X-ray Fluorescence Imaging*: In order to prove the XRF properties of the hybrid Rh-CQDs NPs, a projection image of a sample with a known concentration (200 μg/mL) was acquired. A 2 mL vial was filled with 1 mL sample and introduced into the XRF setup. The step size was set as 200 μm and the exposure time as 10 ms. These settings were already proved to be suitable for tomographic imaging (XFCT) of small animals; the radiation dose for a projection image on a mouse is estimated as 1 mGy. To obtain the final image, the XRF signal (color) was overlaid on top of the X-ray absorption projection (grayscale). Further details about the X-ray source, detectors and setup were described in our previous works [10].

## 3. Results and Discussion

**Figure 1:**
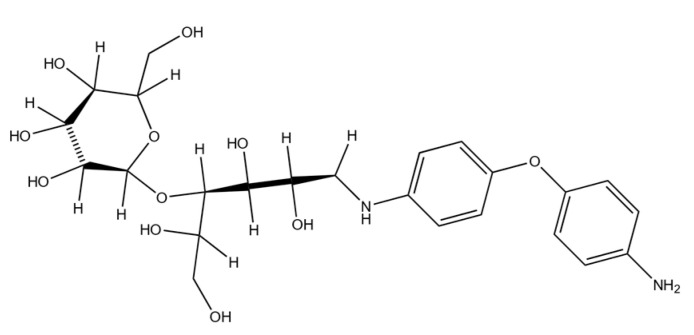
Molecular structure of the synthesized sugar ligand LODAN.

Synthesis of Rh NPs was performed using a specially synthesized sugar ligand, Lactose 4,4′-oxydianiline-N (LODAN), with the detailed molecular structure presented in Figure 1. Its structure is characterized by the presence of multiple hydroxyl groups and one primary amine, which provides LODAN with positive charge at neutral pH. Furthermore, the molecular weight of the sugar ligand was estimated via Mass Spectroscopy (Figure S1a), obtaining 524 ± 1 Da, while the thermogram from thermogravimetric analysis is shown in Figure S1b. A detailed analysis of the thermogram is presented in the Supplementary Materials.

**Figure 2:**
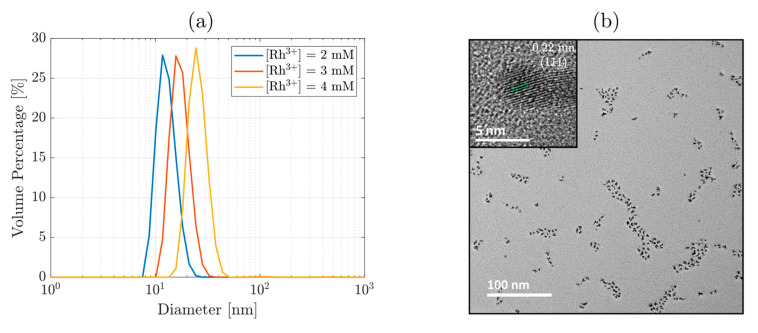
Hydrodynamic size distribution of Rh NPs (**a**), using different Rh precursor concentrations in the synthesis process. (**b**) TEM micrograph of Rh NPs, [Rh^3+^] = 2 mM, containing an insert with HRTEM on a single particle.

The synthesis of Rh NPs was accomplished via a green chemical route, where water was chosen as the solvent for the microwave (MW)-assisted hydrothermal synthesis. As a matter of fact, the synthesis of metallic, Rh-based NPs employing other synthesis methods [12] has made the separation and purification steps nontrivial. The introduction of the sugar ligand, LODAN, provides abundant hydroxyl groups for the reduction reaction of the water-soluble Rh precursor, thus avoiding the use of organic solvents. A series of Rh NPs were synthesized by using varying concentrations of Rh precursor ([Rh^3+^]), while keeping the LODAN concentration and the reaction temperature (160 °C) constant. The hydrodynamic size of the resultant Rh NPs was evaluated as a function of [Rh^3+^] (Figure 2a), where lower concentrations led to smaller NPs, assessed by the hydrodynamic size, indicating the role of capping agent fulfilled by the sugar ligand, besides acting as a reducing agent. A higher [LODAN]/[Rh^3+^] ratio led to smaller NPs. Hence, optimization of this parameter was crucial for an effective control of Rh NP size. By employing 2 mM Rh^3+^, we obtained a hydrodynamic size of 12 ± 3 nm, while for 3 and 4 mM [Rh^3+^], the average NP size was 17 ± 3 and 25 ± 4 nm, respectively. The condition yielding the lowest hydrodynamic size was selected for further use. Morphology and dry size of Rh NPs was analyzed with TEM, and a micrograph is presented in Figure 2b. The NPs were observed to have quasi-spherical morphology with an average diameter of 4 ± 1 nm from the TEM micrographs. The size distribution histogram (Figure S2a) highlighted a lognormal distribution, and the EDS analysis (Figure S2b) confirmed the absence of other elements constituting the Rh NPs. Furthermore, the crystalline nature can be observed in the insert of Figure 2b, where the interplanar distance of 0.22 nm is clearly identified, which corresponds to (111) Bragg diffraction of the cubic close-packed Rh crystal.

Zeta potential measurement on a dispersion of Rh NPs resulted in a strongly positive surface charge (+34 ± 2 mV) at pH 6.5, revealing the presence of protonated amino groups on the NPs’ surface. These amino-functionalized Rh NPs, thus, constitute a robust platform for further surface modifications.

**Figure 3:**
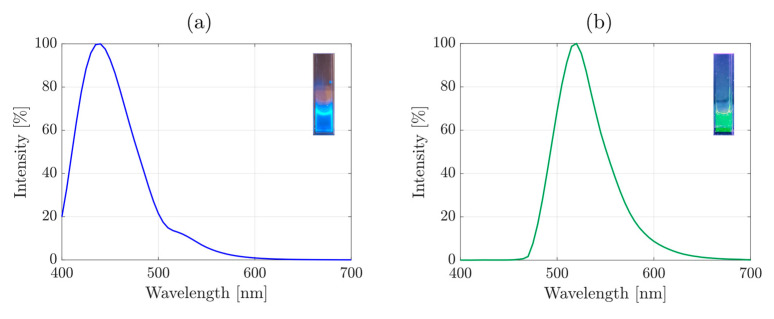
Optical fluorescence (PL) spectra for CQDs (**a**) and Rh-CQDs NPs (**b**) with excitation wavelengths of 390 and 490 nm, respectively. In the inserts, photographs of corresponding samples in a vial irradiated with UV light are presented.

CQDs were synthesized through aqueous pyrolysis of citrate precursor in a basic solution, at the selected reaction temperature of 200 °C for the highest reaction yield (Figure S3), via MW-assisted hydrothermal route. The synthesized CQDs exhibited a narrow lognormal size distribution with an average size of 1.6 ± 0.4 nm as observed from the TEM micrograph (Figure S4a) and their size distribution histogram (Figure S4b). FT-IR analysis highlighted the CQDs’ composition (Figure S5). We further evaluated their fluorescence properties through PL measurements that showed a single peak with emission at 440 nm (blue) and excitation at 390 nm (Figure 3a and Figure S6a). The relative fluorescence Quantum Yield (QY) was estimated as 17%, by comparison with quinine hemisulfate (Table S1), in-line with the earlier reported values for nitrogen-doped CQDs [45]. Further details are described in Supplementary Materials. Furthermore, the absence of multiple peaks and shifts along the emission axis in the 2D fluorescence spectrum proves that the synthesized CQDs displayed excitation-independent emission behavior, which represents a critical parameter for bioimaging, to prevent crosstalk between the different fluorophores [46]. This behavior arises from the highly narrow size distribution, uniformity of surface states and from nitrogen doping of the CQDs [47,48,49,50,51], demonstrated with TEM and FT-IR (Figures S4a and S5). Having confirmed the physicochemical properties of the CQDs, their cytotoxicity was tested in vitro. We exposed RAW 264.7 macrophage cell line to different CQDs concentrations and followed the viability and proliferation of the cells in real time over a period of 60 h (Figure S7a). The Real Time Cell Analysis (RTCA) assay showed a clear concentration-dependent response in RAW264.7 cells exposed to CQDs. After an initial impact on the cell viability, observed between 12 and 24 h, the viability follows a logarithmic increase over time, indicating the absence of long-term cytotoxic effects. The half maximal inhibitory concentration (IC50) was found to be 100 μg/mL, after 36 h exposure of CQDs to the cell culture. The optical fluorescence properties of the CQD phagocyted by macrophages were demonstrated in vitro, with confocal microscopy (Figure S7b), where CQDs could be localized in the cytoplasm already after 3 h exposure, indicating that phagocytosis and transport into the intracellular compartment of cells did not affect the optical properties of the CQDs.

**Figure 4:**
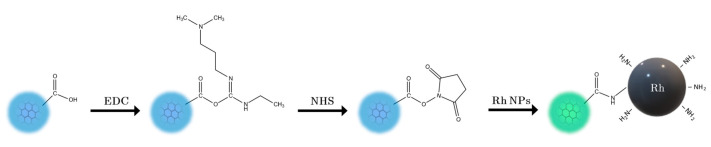
Conjugation scheme of Rh NPs with CQDs, leading to the green-emitting hybrid Rh-CQDs NPs.

Given their biocompatibility and optical fluorescence properties, the CQDs were employed as a passivation coating for X-ray fluorescence (XRF) active contrast agents, Rh NPs, aiming at providing dual fluorescence properties. The synthesized CQDs possess a strong negative surface charge due to surface-exposed carboxyl groups (−33 ± 1 mV at pH 6.5). The carboxyl groups present on the CQDs were used to conjugate them with Rh NPs through the EDC-NHS coupling reaction, obtaining the Rh-CQDs NPs, as schematically shown in Figure 4. Interestingly, the emission band observed for the CQDs shifted to green (520 nm) upon the conjugation process (Figure 3b), due to the surface modification of CQDs when binding Rh NPs. This is mainly attributed to the reduction of pyrrolic nitrogen content on the CQDs (for further details see the Supplementary Materials). Furthermore, the FT-IR spectrum of Rh-CQDs NPs (Figure S8a) highlighted the presence of (C=O) carboxamide stretching vibrations, with an absorption band at 1637 cm^−1^, and of reduced amounts of free amino groups, compared with Rh NPs, with a less intense absorption band for the N-H bending mode (scissoring), at 1500 cm^−1^ [52]. In addition, the full width at half maximum (FWHM) of both CQDs and Rh-CQDs are relatively narrow, respectively 70 and 60 nm, evidencing uniformity of surface states and defects [53]. The decrease in FWHM in Rh-CQDs thus indicates the augmented symmetry of the emission traps formed by surface functional groups. The excitation maximum of 490 nm (Figure S8b) makes the Rh-CQDs suitable for optical confocal imaging with green laser (488 nm). The zeta potential of the hybrid Rh-CQDs NPs was estimated as −28 ± 1 mV, revealing the presence of carboxyl groups on the surface of the hybrid NPs, even after the conjugation process. Moreover, the negative charge ensures high colloidal stability at pH > 6.5. Finally, a TEM micrograph on Rh-CQDs NPs (Figure S9) shows a Rh NP conjugated with CQDs, constituting a passivation coating, and providing the fluorescence properties.

**Figure 5:**
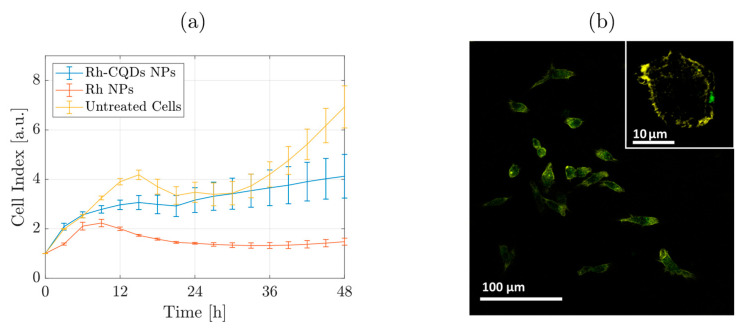
RTCA assay on RAW 264.7 cell lines with Rh and Rh-CQDs NPs (**a**), while keeping [Rh] = 100 μg/mL. The cell index is normalized (*CI* = 1) at the time when NPs were added (*t* = 0). Confocal microscopy images (**b**) of fixed and stained RAW264.7 Macrophages incubated for 24 h with Rh-CQDs (100 μg/mL, in green), at 20× (63× in the insert). Alexa 555 Phalloidin (yellow) is used to visualize the plasma membrane.

The improved biocompatibility of Rh-CQDs NPs with respect to Rh NPs was demonstrated in vitro on RAW264.7 macrophages with the RTCA assay (Figure 5a and Figure S10), while keeping the same concentration of the XRF active element (Rh), respectively at 100 and 200 μg/mL. The results clearly show that the CQDs conjugation with Rh NPs protected the cells from the toxic effect of the core Rh NPs. Both the tested concentrations led to a fatal outcome for the cell culture, when exposed to Rh NPs alone, resulting in a drop in the Cell Index (CI), immediately after the NPs’ introduction. On the contrary, the presence of a CQDs shell, or passivation layer, led to biocompatible hybrid Rh-CQDs NPs, with IC50 of 200 μg/mL after 12 h from the injection.

The intracellular localization of Rh-CQDs NPs in RAW 264 macrophages, after 24 h exposure, was examined using confocal microscopy (Figure 5b). The actin filaments were stained with Alexa 555 Phalloidin, allowing visualization of the cell membrane (in yellow), while the Rh-CQD NPs were detected in the cytoplasm and plasma membrane of the cells, because of their optical fluorescence under excitation at 488 nm (in green).

**Figure 6:**
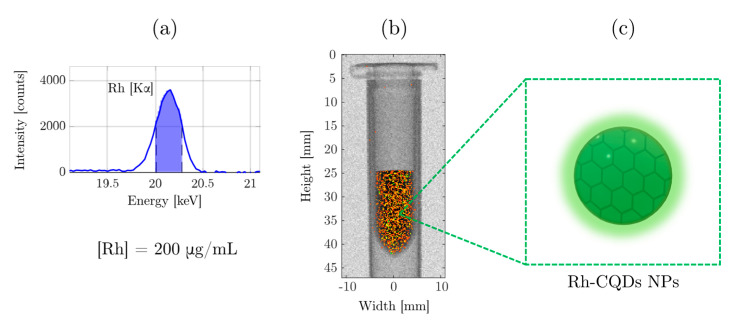
X-ray Fluorescence (XRF) experiment on Rh-CQDs NPs at 200 μg/mL. XRF spectrum recorded for 3 min at the central position of the vial (**a**); projection image of the vial with absorption and XRF signals (**b**); schematic representation of Rh-QDs NPs, contained in the vial (**c**).

Finally, Rh-CQDs NPs were tested for their XRF performance, with a Rh concentration of 200 μg/mL in a phantom experiment. The XRF spectrum (Figure 6a) evidenced the presence of Rh [Kα] peak, centered at 20.1 keV. By scanning the area of the sample with the X-ray pencil beam, the projection image was obtained (Figure 6b), demonstrating the XRF activity of the Rh-CQDs NPs (schematically shown in Figure 6c). The employed concentration was chosen coherently with previous observations of local concentrations of XRF-active NPs in mice [10]. The lower cytotoxicity of the hybrid nanostructures, Rh-CQDs NPs, will hence allow to increase the injected dose in small animals, leading to enhanced XRF detected signal in XFCT, with minimal side-effects. Thus, the improved biocompatibility and the optical fluorescence, granted by the conjugation with CQDs, make these hybrid NPs a potential candidate for in vitro and in vivo contrast agents for diagnostics, empowered by the XRF properties of the Rh NPs combined with the optical fluorescence characteristics of the CQDs.

## 4. Conclusions

A hybrid core-shell NP architecture, combining optical and X-ray fluorescence properties as bioimaging contrast agents is presented. Rh NPs were synthesized through a MW-assisted hydrothermal route by using a custom designed sugar ligand (LODAN), which played a dual function as reducing and surface capping agent. CQDs were synthesized using MW-assisted aqueous pyrolysis of citrate in ammonia solution, yielding large-scale NPs within a significantly short reaction time. The excitation-independent behavior made them suitable for bioimaging, preventing any crosstalk with other dyes. Furthermore, the high photostability and low photobleaching of CQDs constituted the leading reasons for choosing CQDs over organic dyes. Surface exposed amino groups on Rh NPs, due to the LODAN capping, were successfully used to conjugate with CQDs using NHS-EDC click-chemistry. The synthesized sugar ligand, as well as the NPs were evaluated at various processing steps using a library of analytical techniques, including DLS, zeta potential, FT-IR, TGA, TEM, UV-Vis, and PL measurements. CQDs’ conjugation onto Rh NPs resulted in a red shift of the PL of the CQDs, attributed to the surface modification of the CQDs during the EDC-NHS reaction. CQDs provided Rh NPs with optical fluorescence properties and improved their biocompatibility, as demonstrated in vitro by Real-Time Cell Analysis (RTCA) on a macrophage cell line (RAW 264.7). The multimodal fluorescence characteristics of Rh-CQD core-shell NPs are confirmed with confocal microscopy, and XRF phantom experiments. The presented MW-assisted synthetic routes both for Rh NPs and the CQDs are benign, making these materials safe by design, especially when the intended application is for biomedicine.

## Data Availability

Data is available within this manuscript and the Supplementary Material.

## Supplementary Materials

The following are available online at https://www.mdpi.com/article/10.3390/nano11092165/s1, Figure S1: Mass Spectrometry Analysis (a), and Thermogravimetric Analysis (TGA) (b) of the sugar ligand, LODAN. Figure S2: Size distribution histogram (blue) of Rh NPs with a lognormal fit (black) (a). The mean value is highlighted with a dashed line (grey). EDS spectrum of Rh NPs (in TEM) highlighting the Rh emission peaks (b). Figure S3: Optical fluorescence (PL) spectra for CQDs (500 μg/mL), synthesized at different temperatures: 180 °C (green), 200 °C (blue) and 220 °C (red). The intensities were normalized with respect to the highest peak. Figure S4: TEM micrograph of the synthesized CQDs (a) and the size distribution histogram (blue) with a lognormal fit (black) (b). The mean value is highlighted with a dashed line (grey). Figure S5: Infrared Absorption (FT-IR) spectrum of CQDs. Figure S6: 2D Fluorescence spectrum (a), with emission and excitation maxima at 440 nm and 390 nm, respectively, and TGA thermogram (b) of the synthesized CQDs. Nitrogen flow (black) was employed up to 750 °C, substituted by synthetic air flow (red) from 750 °C to 900 °C. Figure S7: RTCA assay on RAW 264.7 cell lines with CQDs (a) at different concentrations (100, 50, 25 and 12.5 μg/mL). The cell index is normalized (*CI* = 1) at the time when NPs were added (t = 0). Confocal microscopy images (b) of fixed and stained RAW264.7 Macrophages incubated for 3 h with CQDs (25 μg/mL, in blue), at 20× (63× in the insert). Alexa 555-Phalloidin (yellow) is used as the marker for actin filaments. Figure S8: Infrared Absorption (FT-IR) Spectra of LODAN (brown), Rh NPs (black), CQDs (blue) and the hybrid Rh-CQDs NPs (green) (a) and 2D Fluorescence spectrum of Rh-CQDs NPs, with emission and excitation maxima at 520 nm and 490 nm (b), respectively. Figure S9: TEM micrograph of a Rh-CQDs hybrid NP. Figure S10: RTCA assay on RAW 264.7 cell lines with Rh-BQDs NPs and Rh NPs, [Rh] = 200 μg/mL The cell index is normalized (*CI* = 1) at the time when NPs were added (*t* = 0). Table S1: Summarized data for absorbance and integrated fluorescence intensity of CQDs and Quinine hemisulfate. References [54,55,56,57,58,59,60,61,62,63,64,65,66,67] are cited in the Supplementary Materials.

## References

[1] Naseri N., Ajorlou E., Asghari F., Pilehvar-Soltanahmadi Y. (2018). An update on nanoparticle-based contrast agents in medical imaging. Artif. Cells Nanomed. Biotechnol..

[2] Peer D., Karp J.M., Hong S., Farokhzad O.C., Margalit R., Langer R. (2007). Nanocarriers as an emerging platform for cancer therapy. Nat. Nanotechnol..

[3] Cheng Z., Al Zaki A., Hui J.Z., Muzykantov V.R., Tsourkas A. (2012). Multifunctional Nanoparticles: Cost Versus Benefit of Adding Targeting and Imaging Capabilities. Science.

[4] Kim J., Chhour P., Hsu J., Litt H.I., Ferrari V.A., Popovtzer R., Cormode D.P. (2017). Use of Nanoparticle Contrast Agents for Cell Tracking with Computed Tomography. Bioconjugate Chem..

[5] Boll H., Nittka S., Doyon F., Neumaier M., Marx A., Kramer M., Groden C., Brockmann M.A. (2011). Micro-CT Based Experimental Liver Imaging Using a Nanoparticulate Contrast Agent: A Longitudinal Study in Mice. PLoS ONE.

[6] Han X., Xu K., Taratula O., Farsad K. (2019). Applications of nanoparticles in biomedical imaging. Nanoscale.

[7] Cheong S.-K., Jones B., Siddiqi A.K., Liu F., Manohar N., Cho S.H. (2010). X-ray fluorescence computed tomography (XFCT) imaging of gold nanoparticle-loaded objects using 110 kVp X-rays. Phys. Med. Biol..

[8] Larsson J.C., Vogt C., Vågberg W., Toprak M.S., Dzieran J., Arsenian-Henriksson M., Hertz H.M. (2018). High-spatial-resolution x-ray fluorescence tomography with spectrally matched nanoparticles. Phys. Med. Biol..

[9] Hertz H.M., Larsson J.C., Lundström U., Larsson D.H., Vogt C. (2014). Laboratory x-ray fluorescence tomography for high-resolution nanoparticle bio-imaging. Opt. Lett..

[10] Shaker K., Vogt C., Katsu-Jimenez Y., Kuiper R.V., Andersson K., Li Y., Larsson J.C., Rodriguez-Garcia A., Toprak M.S., Arsenian-Henriksson M. (2020). Longitudinal In-Vivo X-ray Fluorescence Computed Tomography with Molybdenum Nanoparticles. IEEE Trans. Med. Imaging.

[11] Li Y., Shaker K., Svenda M., Vogt C., Hertz H.M., Toprak M.S. (2020). Synthesis and Cytotoxicity Studies on Ru and Rh Nanoparticles as Potential X-ray Fluorescence Computed Tomography (XFCT) Contrast Agents. Nanomaterials.

[12] Li Y., Saladino G., Shaker K., Svenda M., Vogt C., Brodin B., Hertz H., Toprak M. (2020). Synthesis, Physicochemical Characterization, and Cytotoxicity Assessment of Rh Nanoparticles with Different Morphologies-as Potential XFCT Nanoprobes. Nanomaterials.

[13] Li Y., Shaker K., Larsson J.C., Vogt C., Hertz H.M., Toprak M.S. (2018). A Library of Potential Nanoparticle Contrast Agents for X-ray Fluorescence Tomography Bioimaging. Contrast Media Mol. Imaging.

[14] Saladino G.M., Vogt C., Li Y., Shaker K., Brodin B., Svenda M., Hertz H.M., Toprak M.S. (2021). Optical and X-ray Fluorescent Nanoparticles for Dual Mode Bioimaging. ACS Nano.

[15] Piella J., Bastús N.G., Puntes V. (2016). Size-Controlled Synthesis of Sub-10-nanometer Citrate-Stabilized Gold Nanoparticles and Related Optical Properties. Chem. Mater..

[16] Poda A.R., Kennedy A.J., Cuddy M.F., Bednar A.J. (2013). Investigations of UV photolysis of PVP-capped silver nanoparticles in the presence and absence of dissolved organic carbon. J. Nanoparticle Res..

[17] Kim Y.H., Kang Y.S., Lee W.J., Jo B.G., Jeong J.H. (2006). Synthesis of Cu Nanoparticles Prepared by Using Thermal Decomposition of Cu-oleate Complex. Mol. Cryst. Liq. Cryst..

[18] Pandey P.A., Bell G.R., Rourke J., Sanchez A., Elkin M.D., Hickey B.J., Wilson N.R. (2011). Physical Vapor Deposition of Metal Nanoparticles on Chemically Modified Graphene: Observations on Metal-Graphene Interactions. Small.

[19] Zhang Y., Chen F., Zhuang J., Tang Y., Wang D., Wang Y., Dong A., Ren N. (2002). Synthesis of silver nanoparticles via electrochemical reduction on compact zeolite film modified electrodes. Chem. Commun..

[20] Suslick K., Choe S.-B., Cichowlas A.A., Grinstaff M. (1991). Sonochemical synthesis of amorphous iron. Nat. Cell Biol..

[21] Dahal N., García S., Zhou J., Humphrey S.M. (2012). Beneficial Effects of Microwave-Assisted Heating versus Conventional Heating in Noble Metal Nanoparticle Synthesis. ACS Nano.

[22] Kang S., Shin W., Choi M.-H., Ahn M., Kim Y.-K., Kim S., Min D.-H., Jang H. (2018). Morphology-Controlled Synthesis of Rhodium Nanoparticles for Cancer Phototherapy. ACS Nano.

[23] Compostella F., Pitirollo O., Silvestri A., Polito L. (2017). Glyco-gold nanoparticles: Synthesis and applications. Beilstein J. Org. Chem..

[24] Mocanu A., Cernica I., Tomoaia G., Bobos L.-D., Horovitz O., Tomoaia-Cotisel M. (2009). Self-assembly characteristics of gold nanoparticles in the presence of cysteine. Coll. Surf. A Physicochem. Eng. Asp..

[25] Lee H.-E., Ahn H.-Y., Mun J., Lee Y.Y., Kim M., Cho N.H., Chang K., Kim W.S., Rho J., Nam K.T. (2018). Amino-acid- and peptide-directed synthesis of chiral plasmonic gold nanoparticles. Nat. Cell Biol..

[26] Soliveri G., Ardizzone S., Yüksel S., Cialla-May D., Popp J., Schubert U.S., Hoeppener S. (2016). Microwave-Assisted Silver Nanoparticle Film Formation for SERS Applications. J. Phys. Chem. C.

[27] Katti K.K., Kattumuri V., Bhaskaran S., Katti K.V., Kannan R. (2009). Facile and General Method for Synthesis of Sugar-Coated Gold Nanoparticles. Int. J. Nanotechnol. Biomed..

[28] Darroudi M., Ahmad M.B., Abdullah A.H., Ibrahim N.A. (2011). Green synthesis and characterization of gelatin-based and sugar-reduced silver nanoparticles. Int. J. Nanomed..

[29] Yazgan I., Gümüş A., Gökkuş K., Demir M.A., Evecen S., Sönmez H.A., Miller R.M., Bakar F., Oral A., Popov S. (2020). The Effect of Modified Carbohydrates on the Size and Shape of Gold and Silver Nanostructures. Nanomaterials.

[30] Saladino G.M., Hamawandi B., Demir M.A., Yazgan I., Toprak M.S. (2021). A versatile strategy to synthesize sugar ligand coated superparamagnetic iron oxide nanoparticles and investigation of their antibacterial activity. Coll. Surf. A Physicochem. Eng. Asp..

[31] Zou X., Zhang L., Wang Z., Luo Y. (2016). Mechanisms of the Antimicrobial Activities of Graphene Materials. J. Am. Chem. Soc..

[32] Alaghmandfard A., Sedighi O., Rezaei N.T., Abedini A.A., Khachatourian A.M., Toprak M.S., Seifalian A. (2021). Recent advances in the modification of carbon-based quantum dots for biomedical applications. Mater. Sci. Eng. C.

[33] Xu A., Wang G., Li Y., Dong H., Yang S., He P., Ding G. (2020). Carbon-Based Quantum Dots with Solid-State Photoluminescent: Mechanism, Implementation, and Application. Small.

[34] Zhu S., Song Y., Zhao X., Shao J., Zhang J., Yang B. (2015). The photoluminescence mechanism in carbon dots (graphene quantum dots, carbon nanodots, and polymer dots): Current state and future perspective. Nano Res..

[35] Shen J., Zhu Y., Yang X., Li C. (2012). Graphene quantum dots: Emergent nanolights for bioimaging, sensors, catalysis and photovoltaic devices. Chem. Commun..

[36] Dong J., Wang K., Sun L., Sun B., Yang M., Chen H., Wang Y., Sun J., Dong L. (2018). Application of graphene quantum dots for simultaneous fluorescence imaging and tumor-targeted drug delivery. Sens. Actuators B Chem..

[37] Li K., Liu W., Ni Y., Li D., Lin D., Su Z., Wei G. (2017). Technical synthesis and biomedical applications of graphene quantum dots. J. Mater. Chem. B.

[38] Hassan M., Gomes V.G., Dehghani A., Ardekani S.M. (2018). Engineering carbon quantum dots for photomediated theranostics. Nano Res..

[39] Atabaev T.S. (2018). Doped Carbon Dots for Sensing and Bioimaging Applications: A Minireview. Nanomaterials.

[40] Lin L., Luo Y., Tsai P., Wang J., Chen X. (2018). Metal ions doped carbon quantum dots: Synthesis, physicochemical properties, and their applications. TrAC Trends Anal. Chem..

[41] Zhang J., Liu X., Wang X., Mu L., Yuan M., Liu B., Shi H. (2018). Carbon dots-decorated Na2W4O13 composite with WO3 for highly efficient photocatalytic antibacterial activity. J. Hazard. Mater..

[42] Tejwan N., Saini A.K., Sharma A., Singh T.A., Kumar N., Das J. (2021). Metal-doped and hybrid carbon dots: A comprehensive review on their synthesis and biomedical applications. J. Control. Release.

[43] Seo J.H., Adachi K., Lee B.K., Kang D.G., Kim Y.K., Kim K.R., Lee H.Y., Kawai T., Cha H.J. (2007). Facile and Rapid Direct Gold Surface Immobilization with Controlled Orientation for Carbohydrates. Bioconjugate Chem..

[44] Yazgan İ. (2019). Synthesis of Open-Chain Sugar Derivatives as Anticancer and Antimicrobial Agents. Commun. Fac. Sci. Univ. Ankara Ser. C Biol..

[45] Namdari P., Negahdari B., Eatemadi A. (2017). Synthesis, properties and biomedical applications of carbon-based quantum dots: An updated review. Biomed. Pharmacother..

[46] Dong Y., Pang H., Bin Yang H., Guo C., Shao J., Chi Y., Li C.M., Yu T. (2013). Carbon-Based Dots Co-doped with Nitrogen and Sulfur for High Quantum Yield and Excitation-Independent Emission. Angew. Chem. Int. Ed..

[47] Mikhraliieva A., Zaitsev V., Xing Y., Coelho-Júnior H., Sommer R.L. (2020). Excitation-Independent Blue-Emitting Carbon Dots from Mesoporous Aminosilica Nanoreactor for Bioanalytical Application. ACS Appl. Nano Mater..

[48] Lesani P., Ardekani S.M., Dehghani A., Hassan M., Gomes V.G. (2019). Excitation-independent carbon dot probes for exogenous and endogenous Fe3+ sensing in living cells: Fluorescence lifetime and sensing mechanism. Sens. Actuators B Chem..

[49] Dager A., Uchida T., Maekawa T., Tachibana M. (2019). Synthesis and characterization of Mono-disperse Carbon Quantum Dots from Fennel Seeds: Photoluminescence analysis using Machine Learning. Sci. Rep..

[50] Zhang Y., Hu Y., Lin J., Fan Y., Li Y., Lv Y., Liu X. (2016). Excitation Wavelength Independence: Toward Low-Threshold Amplified Spontaneous Emission from Carbon Nanodots. ACS Appl. Mater. Interfaces.

[51] Bharathi G., Nataraj D., Premkumar S., Saravanan P., Thangadurai D.T., Khyzhun O.Y., Senthilkumar K., Kathiresan R., Kolandaivel P., Gupta M. (2020). Insight into the photophysics of strong dual emission (blue & green) producing graphene quantum dot clusters and their application towards selective and sensitive detection of trace level Fe^3+^ and Cr^6+^ ions. RSC Adv..

[52] Gee C., Douin S., Crépin C., Bréchignac P. (2001). Infrared spectroscopy of aniline (C_6_H_5_NH_2_) and its cation in a cryogenic argon matrix. Chem. Phys. Lett..

[53] Torchynska T., Polupan G., Macotela L.V. (2017). Emission transformation in CdSe/ZnS quantum dots conjugated to biomolecules. J. Photochem. Photobiol. B Biol..

[54] Williams P.T., Besler S. (1993). Thermogravimetric Analysis of the Components of Biomass.

[55] Guo L., Santschi P.H., Wilkinson K.J., Lead J.R. (2006). Ultrafiltration and its Applications to Sampling and Characterisation of Aquatic Colloids. Environmental Colloids and Particles: Behaviour, Separation and Characterisation.

[56] Gu S., Hsieh C.-T., Yuan C.-Y., Gandomi Y.A., Chang J.-K., Fu C.-C., Yang J.-W., Juang R.-S. (2019). Fluorescence of functionalized graphene quantum dots prepared from infrared-assisted pyrolysis of citric acid and urea. J. Lumin..

[57] Liu W., Jia H., Zhang J., Tang J., Wang J., Fang D. (2020). Preparation of nitrogen-doped carbon quantum dots (NCQDs) and application for non-enzymatic detection of glucose. Microchem. J..

[58] Saladino G.M., Hamawandi B., Vogt C., Rajarao G.K., Toprak M.S. (2020). Click chemical assembly and validation of bio-functionalized superparamagnetic hybrid microspheres. Appl. Nanosci..

[59] Dang D.K., Sundaram C., Ngo Y.-L.T., Choi W.M., Chung J.S., Kim E.J., Hur S.H. (2019). Pyromellitic acid-derived highly fluorescent N-doped carbon dots for the sensitive and selective determination of 4-nitrophenol. Dye. Pigment..

[60] Würth C., Grabolle M., Pauli J., Spieles M., Resch-Genger U. (2013). Relative and absolute determination of fluorescence quantum yields of transparent samples. Nat. Protoc..

[61] Williams A.T.R., Winfield S.A., Miller J.N. (1983). Relative fluorescence quantum yields using a computer-controlled luminescence spectrometer. Analyst.

[62] Redshaw C., Elsegood M.R.J., Frese J.W.A., Ashby S., Chao Y., Mueller A. (2012). Cellular uptake studies of two hexanuclear, carboxylate bridged, zinc ring structures using fluorescence microscopy. Chem. Commun..

[63] Şenel B., Demir N., Büyükköroğlu G., Yıldız M. (2019). Graphene quantum dots: Synthesis, characterization, cell viability, genotoxicity for biomedical applications. Saudi Pharm. J..

[64] Tripathy N., Hong T.-K., Ha K.-T., Jeong H.-S., Hahn Y.-B. (2014). Effect of ZnO nanoparticles aggregation on the toxicity in RAW 264.7 murine macrophage. J. Hazard. Mater..

[65] Carlander U., Midander K., Hedberg Y., Johanson G., Bottai M., Karlsson H.L. (2019). Macrophage-Assisted Dissolution of Gold Nanoparticles. ACS Appl. Bio. Mater..

[66] Hermanson G.T. (2013). Bioconjugate Techniques.

[67] Alam Sk M., Ananthanarayanan A., Huang L., Lim K.H., Chen P. (2014). Revealing the tunable photoluminescence properties of graphene quantum dots. J. Mater. Chem. C.

