# Supplementary material for: Carbon Quantum Dots Conjugated Rhodium Nanoparticles as Hybrid Multimodal Contrast Agents
Source: Nanomaterials (Basel). 2021 Aug 24;11(9):2165. doi: 10.3390/nano11092165 (PMC8470909; doi:10.3390/nano11092165)
Supplement: Supplementary file 1 [file nanomaterials-11-02165-s001.zip › nanomaterials-1343385-supplementary.pdf]

nanomaterials

Supplementary Materials

Carbon Quantum Dots Conjugated Rhodium Nanoparticles as Hybrid Multimodal Contrast Agents

Giovanni M. Saladino [1,*], Nuzhet I. Kilic [1], Bertha Brodin [1], Bejan Hamawandi [1], Idris Yazgan [2], Hans M. Hertz [1] and Muhammet S. Toprak [1,*]

1 Department of Applied Physics, Biomedical and X-Ray Physics, KTH Royal Institute of Technology, SE 10691 Stockholm, Sweden; nikilic@kth.se (N.I.K.); berthab@kth.se (B.B.); bejan@kth.se (B.H.); hans.hertz@biox.kth.se (H.M.H.)
2 Center of Biosensors and Materials, Department of Biology, Faculty of Science and Arts, Kastamonu University, 37150 Kastamonu, Turkey; iyazgan@kastamonu.edu.tr
* Correspondence: saladino@kth.se (G.M.S.); toprak@kth.se (M.S.T.)

Citation: Saladino, G.M.; Kilic, N.I.; Brodin, B.; Hamawandi, B.; Yazgan, I.; Hertz, H.M.; Toprak, M.S. Carbon Quantum Dots Conjugated Rhodium Nanoparticles as Hybrid Multimodal Contrast Agents. *Nanomaterials* **2021**, *11*, 2165. https://doi.org/10.3390/nano11092165

Academic Editors: Alexandru Mihai Grumezescu; Oana Gherasim

Received: 31 July 2021
Accepted: 21 August 2021
Published: 24 August 2021

Publisher's Note: MDPI stays neutral with regard to jurisdictional claims in published maps and institutional affiliations.

Copyright: © 2021 by the authors. Licensee MDPI, Basel, Switzerland. This article is an open access article distributed under the terms and conditions of the Creative Commons Attribution (CC BY) license (http://creativecommons.org/licenses/by/4.0/).

Characterization of the Sugar Ligand

The molecular weight of LODAN was estimated via mass spectroscopy (Figure S1a), as 524 ± 1 Da, while the thermogram from TGA analysis is shown in Figure S1b. The decomposition begins at 180 °C, where an inflection point separates water desorption from thermal decomposition. In order to avoid alteration in the ligand structure and preserve its properties, a temperature below 180 °C was employed for the synthesis of Rh NPs. The decomposition of the sugar ligand proceeds with the elimination of hydroxyl groups in three distinct steps (II to IV), up to about 460 °C, due to the different orientation of the hydroxyl groups leading to different resistances towards pyrolysis [1]. The decomposition of the hydroxyl groups was followed by concurrent reactions of polymerization and char formation (V), where the weight loss is less sharp.

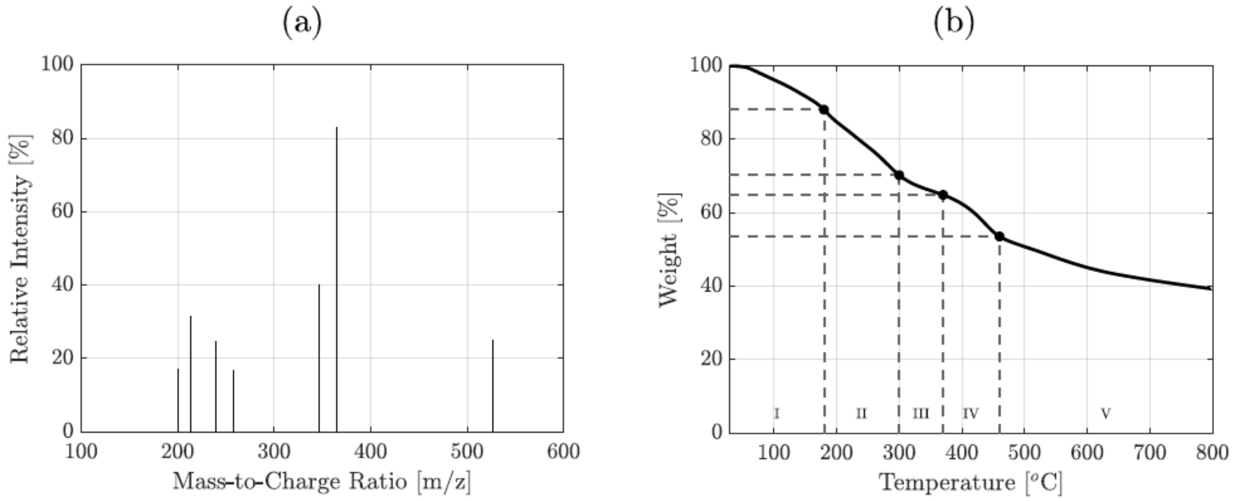

Figure S1. Mass spectrometry analysis (**a**), and Thermogravimetric Analysis (TGA) (**b**) of the sugar ligand, LODAN.

Characterization of the Rhodium Nanoparticles

The diameter of Rh NPs was estimated via TEM micrographs, as 4 ± 1 nm. The size distribution histogram is shown in Figure S2a, together with a lognormal distribution fit (R^2 = 0.99). The lognormal distribution parameters were estimated as μ = 1.37 and σ = 0.31. In Figure S2b, the EDS spectrum obtained of the Rh NPs is plotted. In the scanned energy range, the emission peaks of Rh (Lα, Lβ and Kα) are identified, together with the ones from carbon (C Kα) and copper (Cu Lβ, Cu Kα, Cu Kβ), which are the constituents of the TEM grid (substrate and grid, respectively). The analysis confirmed the absence of other elements in the Rh NPs. Furthermore, the Rh Kα emission peak (20.1 keV) was demonstrated to be optimal for X-ray Fluorescence Computed Tomography (XFCT), with a 25 keV X-ray source [2].

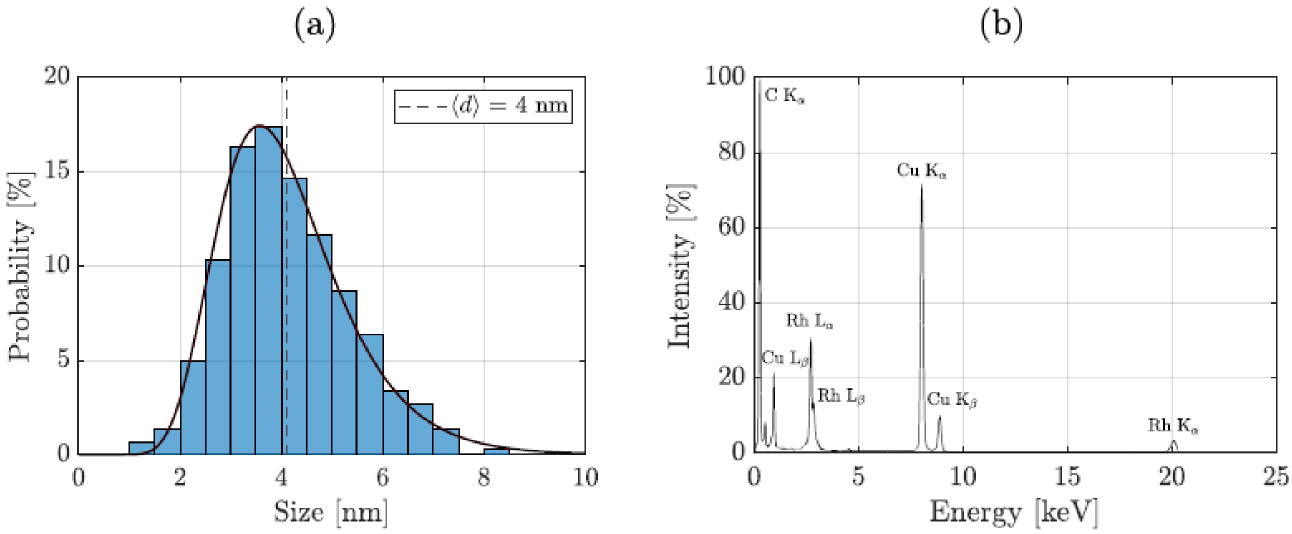

Figure S2. Size distribution histogram (blue) of Rh NPs with a lognormal fit (black) **(a)**. The mean value is highlighted with a dashed line (grey). EDS spectrum of Rh NPs (in TEM) highlighting the Rh emission peaks (**b**).

Characterization of the Carbon Quantum Dots (CQDs)

In order to identify the condition with the highest reaction yield, the effect of the reaction temperature on the fluorescence properties of the sample was investigated. The

reaction solution was processed at three different temperatures (180, 200, and 220 °C) in the MW reactor. Dilutions of the three samples were prepared to obtain the same concentration as 500 µg/mL. In Figure S3, the PL spectra of the three samples are plotted, where the PL intensity correlates linearly with the reaction yield. The low yield at reaction temperature of 180 °C was ascribed to an incomplete reaction, with subsequent presence of unreacted precursor. The reaction performed at 200 °C led to the highest reaction yield. A higher reaction temperature of 220 °C induced carbonization of the citric acid, leading to a lower reaction yield, thus decreased fluorescence intensity. From this series, the optimal reaction temperature of 200 °C was chosen for CQDs synthesis.

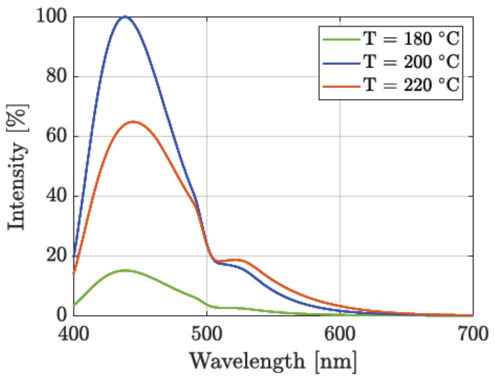

Figure S3. Optical fluorescence (PL) spectra for CQDs (500 µg/mL), synthesized at different temperatures: 180 °C (green), 200 °C (blue), and 220 °C (red). The intensities were normalized with respect to the highest peak.

A typical TEM micrograph for the synthesized CQDs is presented in Figure S4a; their size was estimated as 1.6 ± 0.4 nm. The size distribution histogram is shown in Figure S4b, together with a lognormal distribution fit (R^2 = 0.98). The lognormal distribution parameters were estimated as μ = 0.42 and σ = 0.24. The hydrodynamic size could not be determined with Dynamic Light Scattering due to the fluorescence properties of the CQDs interfering with the scattered light intensity. Instead, a dialysis tube with 3.5 kDa molecular weight cut-off (MWCO) was used; the CQDs passed through the dialysis tube filter confirming the high mono-dispersity and absence of agglomerations, allowing the estimation of an upper-limit for their hydrodynamic size as about 2.68 nm, equal to the cut-off diameter of the 3.5 kDa filter [3]. The fluorescence properties of the synthesized CQDs are studied by obtaining a 2D fluorescence spectrum, shown in Figure S5a. It is possible to identify a single peak in the near UV and visible range, with excitation wavelength between 300 and 600 nm. The excitation peak is found at 390 nm, while the emission maximum is at 440 nm. These characteristic properties make the CQDs suitable for in vitro bioimaging with optical confocal microscopy, by employing a 405 nm laser.

In Figure S5, the FT-IR spectrum of CQDs reveals the presence of carboxyl groups and pyridines on their surface, exhibiting a strong band at 1604 cm^{-1}, which corresponds to the C=O/C=N stretching vibrations [4–6]. The C=C stretching vibration is identified with a sharp peak at 1560 cm^{-1}, while a band centered at 1425 cm^{-1} evidences the presence of primary amides (bending vibration), ascribed to the peptide bond formation between the carboxylic acids present on citric acid and the amines of ammonia solution [5,7]. The weak band at 1185 cm^{-1} corresponds to C–O stretching vibration. Single-bond stretching vibration bands are identifiable at 3428, 3269, and 3030 (2884) cm^{-1}, respectively for O–H, N–H, and C–H stretching vibrations. Lastly, the bands located between 900 and 600 cm^{-1} can be attributed to the N–H bending vibrations, indicating the successful nitrogen doping of CQDs from the ammonia solution, in form of pyrrolic nitrogen and primary amines [7].

The excitation-independent PL behavior, presented in Figure S6a, can be ascribed to highly uniform size distribution and surface states and to nitrogen doping, as

demonstrated in previous studies [8]. The possible aggregation of CQDs in excitation-dependent emission systems could be prevented by separation of larger particles via chromatography and dialysis [9]. The reaction conditions granted by the MW-assisted hydrothermal synthesis, with highly controllable temperature and pressure, could grant high uniformity in the formed products, leading to the absence of an excitation-dependent behavior in the synthesized CQDs [10]. Regarding the uniformity of surface states, the predominance of C=C, C=O and nitrogen-containing functional groups over single bonds (C–O–H and C–O–C) was shown to induce the excitation-independent behavior, due to the lack of emissive traps generated by oxygen-rich functional groups [11–13]. For the determination of the relative fluorescence Quantum Yield (QY), the following formula was utilized [14,15]:

$$QY = QY_r \frac{A_r}{A} \frac{E}{E_r} \left(\frac{n}{n_r}\right)^2 \quad (1)$$

where A is the absorbance of the prepared dispersion (at 390 nm), E is the integrated fluorescence intensity, with the sample excited at 390 nm, n is the refractive index of the solvent, and the subscript r refers to the reference, quinine hemisulfate. The CQDs were dispersed in water (n = 1.333), while quinine hemisulfate (QY_r = 54.6 %) was dispersed in sulfuric acid (0.5 M, n_r = 1.346) [16]. The fluorescence intensity was integrated between 400 and 800 nm. The values for the absorbance and integrated fluorescence intensity are reported in Table S1, leading to the estimation of the QY as 17 %. This value is in-line with the earlier reported values for nitrogen-doped CQDs [17].

Table S1. Summarized data for absorbance and integrated fluorescence intensity of CQDs and Quinine hemisulfate.

	**Absorbance (*A*)**	**Fluorescence Intensity (*E*)**
Quinine Hemisulfate	0.0177	13776
CQDs	0.0309	7658

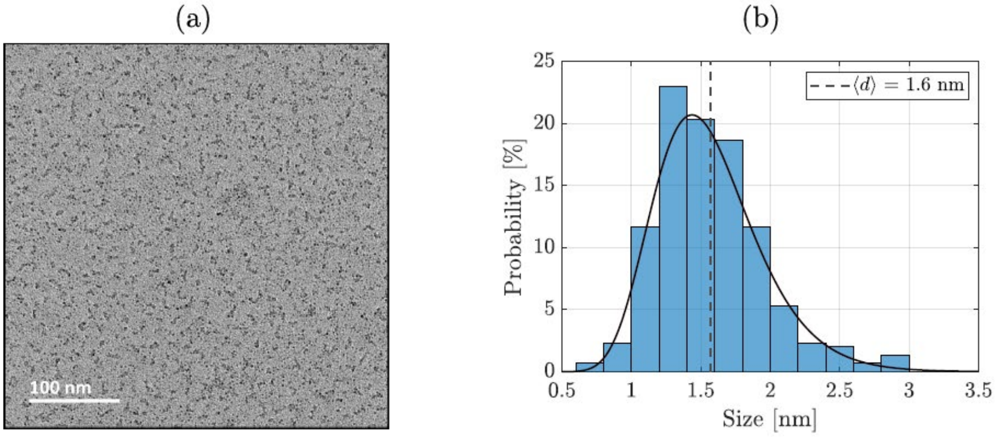

Figure S4. TEM micrograph of the synthesized CQDs **(a)** and the size distribution histogram (blue) with a lognormal fit (black) **(b)**. The mean value is highlighted with a dashed line (grey).

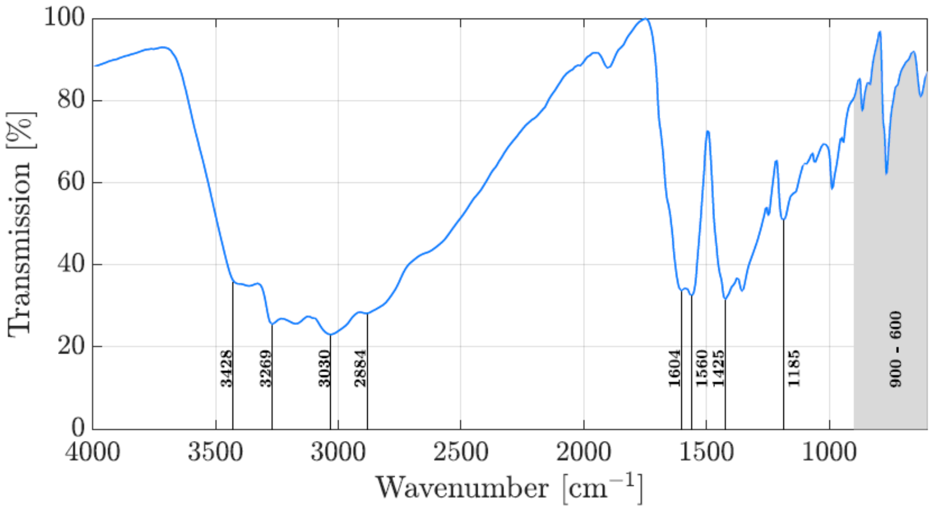

Figure S5. Infrared Absorption (FT-IR) spectrum of CQDs.

Thermogravimetric Analysis (TGA) on concentrated colloidal CQDs, performed under nitrogen flow, reveals details about their composition (Figure S6b). Under nitrogen flow, water desorption is detected up to 180 °C (step I), where an inflection point characterizes the beginning of the decomposition of functional groups on the CQDs. The decomposition is split into two steps (II and III), reaching a steady state at 750 °C. Step II, between 180 and 270 °C, was associated with the decomposition of oxygen-containing groups, such as hydroxyl, carboxyl, amino, and amide groups, present on the CQDs' surface. Subsequently, between 270 and 750 °C, Step III was assigned to the decomposition of pyrrolic and pyridinic nitrogen [18]. Above 750 °C, air flow was introduced for carbon decomposition (step IV), forming carbon oxide by reaction with oxygen (volatile). It was thus possible to estimate the carbon content in the CQDs, as 27 %. Furthermore, the concentration of CQDs was estimated by drying 1 mL of the stock solution (30 °C in vacuum, overnight) and weighing the dried amount. Water content (18 %), estimated via TGA, was subtracted from the measured weight. A typical concentration of about 35 mg/mL was obtained for the stock solution of CQDs.

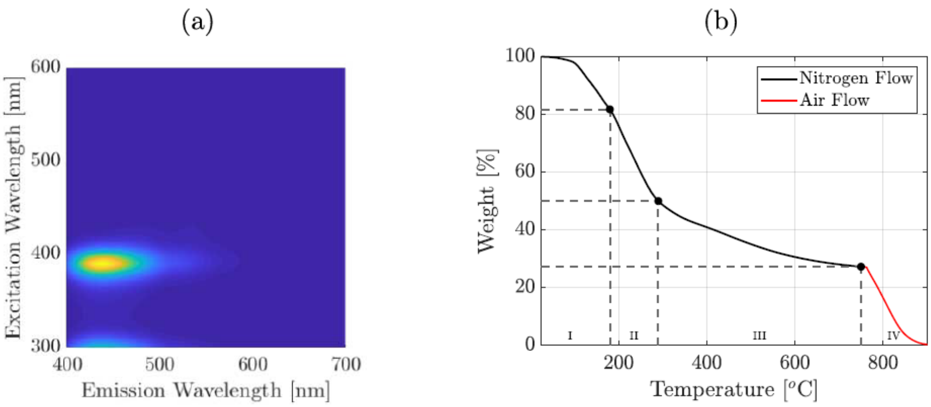

Figure S6. 2D Fluorescence spectrum (**a**), with emission and excitation maxima at 440 and 390 nm, respectively, and TGA thermogram (**b**) of the synthesized CQDs. Nitrogen flow (black) was employed up to 750 °C, substituted by synthetic air flow (red) from 750 to 900 °C.

Cytotoxicity studies are fundamental for newly synthesized NPs, designed for biomedical applications. Although a translation of in vitro results into in vivo scenario is not simple, it is important to select the most significative combination of cell lines and assays.

The Real-Time Cell Analysis (RTCA) assay was employed for these studies, able to monitor the cell behavior as a function of time, without any human intervention. The measurement of the impedance of the electric current flow in the wells permits to obtain a quantity that is directly proportional to the cell confluency, expressed as cell index (*CI*). Phenomena affecting the cell behaviors, such as interrupted proliferation of change in their morphology, would translate in variations in the *CI* [19,20]. Previously conducted studies highlighted interference of Rh NPs with absorption/fluorescence-based cytotoxicity assays (data not shown); hence, the RTCA assay represented a fundamental tool to estimate the effects of the NPs in vitro, allowing the comparison of different kinds of NPs.

A murine macrophage cell line (RAW 264.7) was chosen to test the NPs' biocompatibility. Macrophages, indeed, are of the main components of the immune system, able to identify, engulf, and digest foreign material. The CQDs were thus tested at different concentrations (Figure S7a). Due to their endocytic abilities, macrophages engulf and tend to confine NPs in specific compartments [21], allowing to study the fluorescence properties of the CQDs in vitro, in the cell environment. Confocal microscopy permitted to localize the CQDs in the intracellular environment, by employing a 405 nm laser (Figure S7b), even after only 3 h incubation time with CQDs.

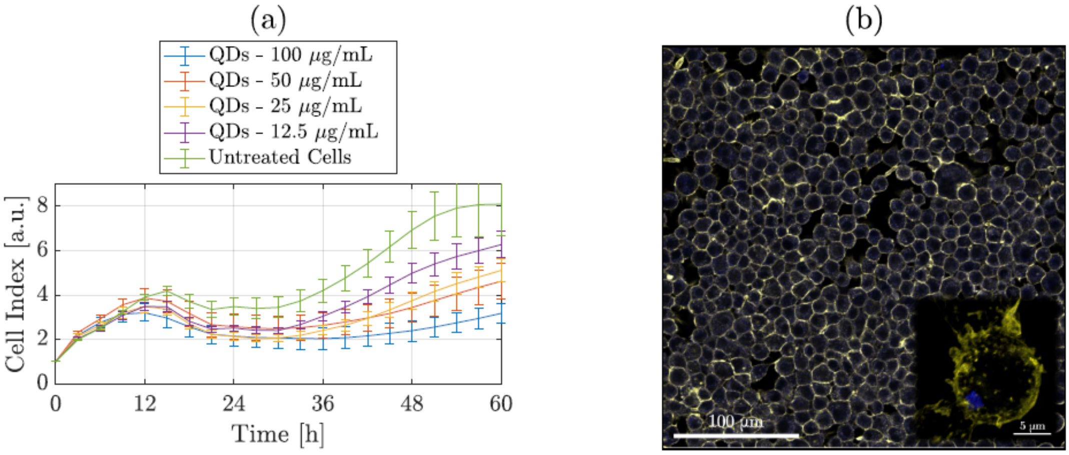

Figure S7. RTCA assay on RAW 264.7 cell lines with CQDs (**a**) at different concentrations (100, 50, 25, and 12.5 μg/mL). The cell index is normalized (*CI* = 1) at the time when NPs were added (*t* = 0). Confocal microscopy images (**b**) of fixed and stained RAW264.7 macrophages incubated for 3 h with CQDs (25 μg/mL, in blue), at 20× (63× in the insert). Alexa 555 - Phalloidin (yellow) was used as the marker for actin filaments.

Characterization of the Conjugated Rhodium NPs with Carbon Quantum Dots

In Figure S8a, the Infrared Absorption (FT-IR) Spectrum of LODAN highlight the presence of free amino groups, through the distinct band for the N–H bending mode (scissoring) at 1500 cm^{-1}, characteristic for amines bonded to phenyl groups (aromatic amine) [22], as visible in the molecular structure (Figure 1). This band is also detectable in the synthesized Rh NPs, which confirms the presence of free amine groups on the Rh NPs' surface, together with the strong positive charge, with their zeta potential of +34 ± 2 mV.

In Rh-CQDs NPs, the stretching vibration band of the carboxyl groups is replaced by a strong band at 1637 cm^{-1}, corresponding to the (C=O) carboxamide stretching, demonstrating the success of the conjugation process. Furthermore, the N–H bending mode (scissoring) of the aromatic amines is strongly weakened in intensity, at 1500 cm^{-1}. Although Rh NPs contain an excess number of amino groups, CQDs also contain nitrogen species such as pyrrolic nitrogen, pyridinic nitrogen, and primary amines, due to the doping (Figure S8a). Thus, the conjugation process also induced the binding of the secondary amines of the CQDs (pyrrolic nitrogen) with the carboxyl groups to form imide groups, although the reaction rate is slower [23]. An increase in the overall size and the reduction in pyrrolic nitrogen as a secondary consequence of the NHS-EDC reaction both contributed to the

redshift of the emission wavelength [24], as shown in the 2D fluorescence spectrum (Figure S8b). The excitation maximum of 490 nm makes the Rh-CQDs suitable for optical confocal imaging with green laser (488 nm), exhibiting a strong emission at 520 nm.

In Figure S9, the TEM micrograph on a single Rh-CQDs NP is presented, highlighting a Rh NP overlapping with CQDs, leading to a passivation coating, and providing fluorescence properties. Although it was not possible to reveal/show the interplanar distance of Rh when defocusing in order to also visualize the carbon shell, the difference in absorption/scattering between the core and the shell can be attributed to the inner Rh NP and surrounding CQDs, respectively. The passivation property is confirmed with RTCA assay, comparing Rh NPs and Rh-CQDs NPs with the same Rh concentration. In Figure S10, a concentration of 200 µg/mL is used to show the drastic behavioral change in cells: whilst Rh NPs led to immediate cell death and detachment from the bottom of the well, Rh-CQDs NPs mainly affected the *CI* after few hours from the incubation, showing a positive slope throughout the whole analysis duration.

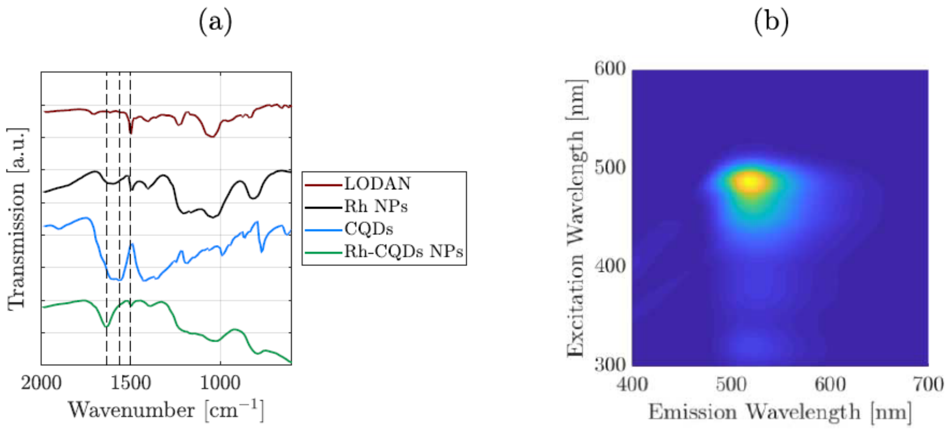

Figure S8. Infrared Absorption (FT-IR) Spectra of LODAN (brown), Rh NPs (black), CQDs (blue) and the hybrid Rh-CQDs NPs (green) (**a**), and 2D Fluorescence spectrum of Rh-CQDs NPs, with emission and excitation maxima at 520 and 490 nm (**b**), respectively.

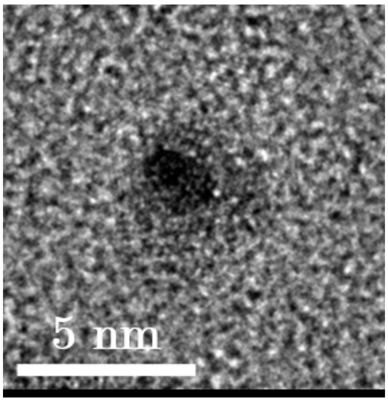

Figure S9. TEM micrograph of a Rh-CQDs hybrid NP.

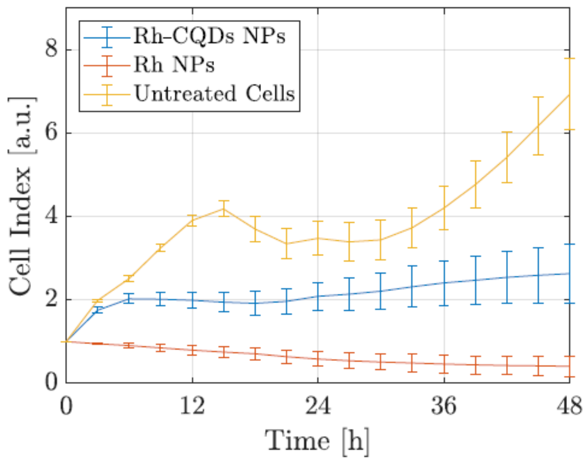

Figure S10. RTCA assay on RAW 264.7 cell lines with Rh-BQDs NPs and Rh NPs, [Rh] = 200 µg/mL. The cell index is normalized (*CI* = 1) at the time when NPs were added (*t* = 0).

References

1. Williams, P.T.; Besler, S. *Thermogravimetric Analysis of the Components of Biomass*. Springer: Dordrecht, Netherlands, 1993, 771–783, doi:10.1007/978-94-011-1336-6_60.
2. Li, Y.; Shaker, K.; Larsson, J.C.; Vogt, C.; Hertz, H.M.; Toprak, M.S. A Library of Potential Nanoparticle Contrast Agents for X-Ray Fluorescence Tomography Bioimaging. *Contrast Media Mol. Imaging* **2018**, *2018*, 1–7, doi:10.1155/2018/8174820.
3. Guo, L.; Santschi, P.H. Ultrafiltration and its Applications to Sampling and Characterisation of Aquatic Colloids. In *Environmental Colloids and Particles: Behaviour, Separation and Characterisation*, Wilkinson, K.J., Lead, J.R., Eds., United Kingdom, 2006, Volume 10, 159–221, doi:10.1002/9780470024539.ch4.
4. Gu, S.; Hsieh, C.-T.; Yuan, C.-Y.; Gandomi, Y.A.; Chang, J.-K.; Fu, C.-C.; Yang, J.-W.; Juang, R.-S. Fluorescence of functionalized graphene quantum dots prepared from infrared-assisted pyrolysis of citric acid and urea. *J. Lumin.* **2019**, *217*, 116774, doi:10.1016/j.jlumin.2019.116774.
5. Liu, W.; Jia, H.; Zhang, J.; Tang, J.; Wang, J.; Fang, D. Preparation of nitrogen-doped carbon quantum dots (NCQDs) and application for non-enzymatic detection of glucose. *Microchem. J.* **2020**, *158*, 105187, doi:10.1016/j.microc.2020.105187.
6. Saladino, G.M.; Hamawandi, B.; Vogt, C.; Rajarao, G.K.; Toprak, M.S. Click chemical assembly and validation of bio-functionalized superparamagnetic hybrid microspheres. *Appl. Nanosci.* **2020**, *10*, 1861–1869, doi:10.1007/s13204-020-01274-5.
7. Dang, D.K.; Sundaram, C.; Ngo, Y.-L.T.; Choi, W.M.; Chung, J.S.; Kim, E.J.; Hur, S.H. Pyromellitic acid-derived highly fluorescent N-doped carbon dots for the sensitive and selective determination of 4-nitrophenol. *Dye. Pigment.* **2019**, *165*, 327–334, doi:10.1016/j.dyepig.2019.02.029.
8. Dong, Y.; Pang, H.; Bin Yang, H.; Guo, C.; Shao, J.; Chi, Y.; Li, C.M.; Yu, T. Carbon-Based Dots Co-doped with Nitrogen and Sulfur for High Quantum Yield and Excitation-Independent Emission. *Angew. Chem. Int. Ed.* **2013**, *52*, 7800–7804, doi:10.1002/anie.201301114.
9. Mikhraliieva, A.; Zaitsev, V.; Xing, Y.; Coelho-Júnior, H.; Sommer, R.L. Excitation-Independent Blue-Emitting Carbon Dots from Mesoporous Aminosilica Nanoreactor for Bioanalytical Application. *ACS Appl. Nano Mater.* **2020**, *3*, 3652–3664, doi:10.1021/acsanm.0c00363.
10. Lesani, P.; Ardekani, S.M.; Dehghani, A.; Hassan, M.; Gomes, V.G. Excitation-independent carbon dot probes for exogenous and endogenous Fe3+ sensing in living cells: Fluorescence lifetime and sensing mechanism. *Sensors Actuators B: Chem.* **2019**, *285*, 145–155, doi:10.1016/j.snb.2019.01.046.
11. Dager, A.; Uchida, T.; Maekawa, T.; Tachibana, M. Synthesis and characterization of Mono-disperse Carbon Quantum Dots from Fennel Seeds: Photoluminescence analysis using Machine Learning. *Sci. Rep.* **2019**, *9*, 1–12, doi:10.1038/s41598-019-50397-5.
12. Zhang, Y.; Hu, Y.; Lin, J.; Fan, Y.; Li, Y.; Lv, Y.; Liu, X. Excitation Wavelength Independence: Toward Low-Threshold Amplified Spontaneous Emission from Carbon Nanodots. *ACS Appl. Mater. Interfaces* **2016**, *8*, 25454–25460, doi:10.1021/acsami.6b08315.
13. Bharathi, G.; Nataraj, D.; Premkumar, S.; Saravanan, P.; Thangadurai, D.T.; Khyzhun, O.Y.; Senthilkumar, K.; Kathiresan, R.; Kolandaivel, P.; Gupta, M.; et al. Insight into the photophysics of strong dual emission (blue & green) producing graphene quantum dot clusters and their application towards selective and sensitive detection of trace level Fe^{3+} and Cr^{6+} ions. *RSC Adv.* **2020**, *10*, 26613–26630, doi:10.1039/d0ra04549g.
14. Würth, C.; Grabolle, M.; Pauli, J.; Spieles, M.; Resch-Genger, U. Relative and absolute determination of fluorescence quantum yields of transparent samples. *Nat. Protoc.* **2013**, *8*, 1535–1550, doi:10.1038/nprot.2013.087.
15. Williams, A.T.R.; Winfield, S.A.; Miller, J.N. Relative fluorescence quantum yields using a computer-controlled luminescence spectrometer. *Analyst* **1983**, *108*, 1067–1071, doi:10.1039/an9830801067.

16. Redshaw, C.; Elsegood, M.R.J.; Frese, J.W.A.; Ashby, S.; Chao, Y.; Mueller, A. Cellular uptake studies of two hexanuclear, carboxylate bridged, zinc ring structures using fluorescence microscopy. *Chem. Commun.* **2012**, *48*, 6627, doi:10.1039/c2cc32060f.
17. Namdari, P.; Negahdari, B.; Eatemadi, A. Synthesis, properties and biomedical applications of carbon-based quantum dots: An updated review. *Biomed. Pharmacother.* **2017**, *87*, 209–222, doi:10.1016/j.biopha.2016.12.108.
18. Şenel, B.; Demir, N.; Büyükköroğlu, G.; Yıldız, M. Graphene quantum dots: Synthesis, characterization, cell viability, genotoxicity for biomedical applications. *Saudi Pharm. J.* **2019**, *27*, 846–858, doi:10.1016/j.jsps.2019.05.006.
19. Tripathy, N.; Hong, T.-K.; Ha, K.-T.; Jeong, H.-S.; Hahn, Y.-B. Effect of ZnO nanoparticles aggregation on the toxicity in RAW 264.7 murine macrophage. *J. Hazard. Mater.* **2014**, *270*, 110–117, doi:10.1016/j.jhazmat.2014.01.043.
20. Carlander, U.; Midander, K.; Hedberg, Y.; Johanson, G.; Bottai, M.; Karlsson, H.L. Macrophage-Assisted Dissolution of Gold Nanoparticles. *ACS Appl. Bio Mater.* **2019**, *2*, 1006–1016, doi:10.1021/acsabm.8b00537.
21. Saladino, G.M.; Vogt, C.; Li, Y.; Shaker, K.; Brodin, B.; Svenda, M.; Hertz, H.M.; Toprak, M.S. Optical and X-ray Fluorescent Nanoparticles for Dual Mode Bioimaging. *ACS Nano* **2021**, *15*, 5077–5085, doi:10.1021/acsnano.0c10127.
22. Gee, C.; Douin, S.; Crépin, C.; Bréchignac, P. Infrared spectroscopy of aniline ($C_6H_5NH_2$) and its cation in a cryogenic argon matrix. *Chem. Phys. Lett.* **2001**, *338*, 130–136, doi:10.1016/s0009-2614(01)00235-4.
23. Hermanson,G.T., *Bioconjugate Techniques*, Third Edition, Academic Press: Cambridge, MA, USA, 2013.
24. Alam Sk, M.; Ananthanarayanan, A.; Huang, L.; Lim, K.H.; Chen, P. Revealing the tunable photoluminescence properties of graphene quantum dots. *J. Mater. Chem. C* **2014**, *2*, 6954–6960, doi:10.1039/c4tc01191k.
